# Comments on Immunogenicity Associated with Botulinum Toxin Treatment. *Toxins* 2019, *11*, 491

**DOI:** 10.3390/toxins12020071

**Published:** 2020-01-23

**Authors:** Keith Foster, Matthew Beard

**Affiliations:** Ipsen Biopharm Ltd., 102 Park Drive, Milton Park, Abingdon, Oxford OX14 4RY, UK; drkafoster@gmail.com

**Keywords:** abobotulinumtoxin A, botulinum neurotoxin (BoNT), immunogenicity, incobotulinumtoxin A, neutralising antibody (NAb), onabotulinumtoxin A, secondary non-responsiveness (SNR)

## Abstract

In contrast to the prevailing arguments presented in the current review, the incidence of neutralising antibody (NAb) formation is not a significant issue for any of the present type A therapeutic botulinum neurotoxin (BoNT) products. Furthermore, clinical non-responsiveness is poorly correlated with the presence of NAbs. The overriding evidence supports the view that the rate of NAb formation is low, does not differ significantly between the different type A BoNT products and that it is not the major factor in clinical response. BoNT products are highly effective and important therapies for the treatment of a variety of neurological and non-neurological conditions.

The manuscript in question seeks to review the role of immunogenicity and the formation of neutralising antibodies (NAbs) in secondary non-responsiveness (SNR) to botulinum neurotoxin (BoNT) treatment, and to relate this to factors such as dosing interval, dose per injection, presence of antigenic protein and the formulation of BoNT product used. A major issue with assessing the role of NAbs in SNR, which the review itself highlights, is the poor relationship between detection of NAbs and SNR. The authors themselves note that: ‘patients with SNR often have no NAbs’ [1], ‘analyses of patients with SNR have shown NAbs prevalence of only 53.5% [2] and 44.5% [3] respectively in two different studies’. The authors also note that: ‘studies looking only at clinically responsive patients have found an even higher prevalence of NAbs … despite continued clinical response’ [4,5]. Despite this uncertainty about the exact relationship between NAbs and SNR, which the authors discuss at length, they frequently nevertheless use data based upon measurement of responsiveness to infer conclusions about the relative immunogenicity of different BoNT products and speculate about factors that might potentially have influenced this. Section 6 of the review, entitled ‘Clinical Resistance Tests’ describes what it denotes as ‘clinical tests of immunoresistance’, specifically the unilateral brow injection (UBI) test, the frontalis antibody test (FTAT), the extensor digitorum brevis (EDB) test, the sternocleidomastoid (SCM) test, and the ninhydrin sweat test (NST). These tests as described, however, assess clinical responsiveness and not immunoresistance. Indeed, as noted in the review itself, there is poor correlation between non-responsiveness in these tests and detection of NAbs. Despite this, the review uses results from these tests to draw conclusions regarding immunogenicity.

The review clearly notes that a key factor influencing the formation of NAbs is dosing interval, and that shorter dosing intervals is the most important risk factor for the formation of NAbs. Despite this, the review advances a view that shorter dosing intervals would be preferable to maintain patient benefit and relates this to the concept of the development of BoNT preparations with lower risk of immunogenicity to enable more frequent dosing. In the context of this discussion the review speculates that incobotulinumtoxin A may be less immunogenic than other available products but omits to discuss how this suggestion could be compatible with the strong evidence suggesting there is no significant difference in the rates of NAb formation between the present type A therapeutic BoNT products [6]. The review also omits to discuss the value of BoNT preparations that maintain patient benefit for longer and enable longer intervals between injection cycles, as recently discussed by Field et al. [7].

A key aspect of the review is to seek to compare the different commercial BoNT products in regard to their relative risk of formation of NAbs. A key reference used to support this perspective is the recent publication by Albrecht et al. [4], in particular the comment that there was ‘14% frequency of NAbs in the study by Albrecht in which the majority of the 596 patients were treated with abobotulinumtoxin A’. This is despite the fact that earlier in the review it had been noted in respect of the Albrecht et al. publication that ‘The authors, however, provided no data on the correlation between the presence of antibodies … and clinical response’. They also noted that ‘their patients were still responding’. In other words, as in other studies, there is no correlation in Albrecht between measuring NAbs and clinical response. The Albrecht study is deeply flawed, and has been previously criticised, not least by one of the current authors, who stated: ‘they failed to mention other studies showing much lower frequency of such antibodies’ and ‘Albrecht et al. provided no data on the correlation between the presence of antibodies detected by mouse hemidiaphragm assay and clinical response’ [8]. Mathevon et al. [9] also commented in review of the Albrecht study that ‘the comparison of formulations was difficult given the different treatment durations for different indications’. One conclusion given in the review is that incobotulinumtoxin A is less immunogenic, and more suited to use with shorter dosing intervals, than other BoNT products because of the lack of neurotoxin associated proteins (NAPs). Reference is also made to Dressler et al. [10] who reported that, because of its lower immunogenicity, incobotulinumtoxin A can be used in patients who have previously developed NAbs and SNR to other BoNT products. This study, however, only examined patients retreated with incobotulinumtoxin A and failed to assess whether the patients would have also responded to re-treatment with the other BoNT products, also, as noted by Bellows and Jankovic, the study was based upon only eight patients, making it difficult to draw meaningful conclusions. The authors of the current review again fail to note that the predominant view drawn from a number of recent systematic reviews across large numbers of studies is that there is no significant difference in the immunogenicity rates observed with the three main type A BoNT products (abobotulinumtoxin A, incobotulinumtoxin A and onabotulinumtoxin A). These conclusions are evident in a number of the references cited in the current manuscript [5,6], and also others that were omitted by the authors [9]. The overriding conclusion from these studies is that immunogenicity is not a significant issue with the main type A BoNT products, and that there is no significant difference between the products in the immunogenicity rates observed. The most comprehensive recent systematic review on this subject by Lacroix-Desmazes and colleagues [6] reviewed a total of 5811 subjects across 31 studies from 1968 to 2013, and found that the rate of NAb development was 1.4% with abobotulinumtoxin A, 0.8–1.1% with incobotulinumtoxin A, and 3.6% with the current onabotulinumtoxin A. The overall prevalence across all studies was 1.9%.

Among the factors discussed in the current review as potential sources of immunogenicity differences between the products are the presence or absence of NAPs, and the ratio of inactive to active neurotoxin protein in the preparation. With regard to complexing proteins, however, the main support provided is from a selection of articles demonstrating a ‘relatively low frequency of antibody formation in patients treated with incobotulinumtoxin A’. These articles, however, include the study of Albrecht, which as discussed above has serious flaws in its design and is not supported by a systematic review across a range of published studies on the subject [6]. They also quote the review of Fabbri et al. [2], but this review did not provide figures for the incidence of NAbs in all patients treated with the different BoNT products, rather separately considering responding and SNR patients. Furthermore, as already discussed and acknowledged in the current review and Fabbri et al. the relationship between SNR and NAb formation is not clear. As discussed above, the overriding evidence from recent systematic reviews supports the view that there is no significant difference in NAb formation rates between abobotulinumtoxin A, incobotulinumtoxin A and onabotulinumtoxin A, and does not indicate a role for NAPs in this. Regarding the idea that there are differences between the products in the ratio of active to inactive neurotoxin, there is no evidence provided to support this statement. In particular, the suggestion that this reflects different levels of single chain 150 kDa neurotoxin, which has not been activated, is not supported by evidence. The reference given to support this statement [11] makes no reference to unactivated single chain protein, and the comparisons made in that reference are based on total clostridial protein rather than BoNT content and use mouse units to compare activity based upon a fixed conversion ratio, as such the conclusions drawn concerning differences in specific activities between the products are invalid.

In conclusion, Bellows and Jankovic provide an inconsistent and poorly supported view on the relative rates of NAb formation between the different BoNT products and confuse their arguments by inter-relating the occurrence of SNR and NAb formation, which are poorly related phenomena. As noted by the authors themselves, the overriding evidence supports the view that SNR is caused by a large number of factors, including insufficient dose, inappropriate muscle selection, improper injection technique, natural progression of the underlying disease and complications due to prolonged abnormal posture. To the extent that NAb formation is an issue in clinical response, it is a minor one, occurring in only a small percentage of patients, and NAb formation rates do not appear to vary between products. Overall, BoNT therapy is a highly effective and important therapy for the treatment of a variety of neurological and non-neurological conditions.
